# Conditioned Medium from Placental Mesenchymal Stem Cells Reduces Oxidative Stress during the Cryopreservation of *Ex Vivo* Expanded Umbilical Cord Blood Cells

**DOI:** 10.1371/journal.pone.0165466

**Published:** 2016-10-25

**Authors:** Darshana Kadekar, Sonal Rangole, Vaijayanti Kale, Lalita Limaye

**Affiliations:** Stem Cell Laboratory, National Centre for Cell Science, NCCS complex, University of Pune Campus, Ganeshkhind, Pune, 411007, Maharashtra, India; Indian Institute of Toxicology Research, INDIA

## Abstract

**Background:**

The limited cell dose in umbilical cord blood (UCB) necessitates ex *vivo* expansion of UCB. Further, the effective cryopreservation of these expanded cells is important in widening their use in the clinics. During cryopreservation, cells experience oxidative stress due to the generation of reactive oxygen species (ROS). Conditioned medium from mesenchymal stem cells (MSCs-CM) has been shown to alleviate the oxidative stress during wound healing, Alzheimer’s disease and ischemic disease. This premise prompted us to investigate the influence of MSCs-CM during cryopreservation of expanded UCB cells.

**Methodology/Principle findings:**

CM-was collected from cord/placental MSCs(C-MSCs-CM, P-MSC-CM). UCB CD34^+^cells were expanded as suspension cultures in serum free medium containing cytokines for 10 days. Cells were frozen with/without C-MSCs-CM and or P-MSCs-CM in the conventional freezing medium containing 20%FCS +10%DMSO using a programmable freezer and stored in liquid nitrogen. Upon revival, cells frozen with MSCs-CM were found to be superior to cells frozen in conventional medium in terms of viability, CD34^+^content and clonogenecity. Priming of revived cells for 48 hrs with MSCs-CM further improved their transplantation ability, as compared to those cultured without MSCs-CM. P-MSCs-CM radically reduced the oxidative stress in cryopreserved cells, resulting in better post thaw functionality of CD34^+^ cells than with C-MSCs-CM. The observed cryoprotective effect of MSCs-CM was primarily due to anti-oxidative and anti-apoptotic properties of the MSCs-CM and not because of the exosomes secreted by them.

**Conclusions/Significance:**

Our data suggest that MSCs-CM can serve as a valuable additive to the freezing or the priming medium for expanded UCB cells, which would increase their clinical applicability.

## Introduction

Umbilical cord blood (UCB) has been widely used as a source of hematopoietic stem cells (HSCs) for the treatment of acquired and hereditary diseases of the hematopoietic system [1–3]. However, insufficient numbers of HSCs in a single UCB unit limits its application especially in adult patients. Thus, *ex vivo* expansion of UCB CD34^+^ cells is required to enable the use of such low cell dose CB units. Many investigators have optimized the conditions for expanding HSCs without deteriorating their capacity to provide a lifelong supply of blood cells post transplantation as is reflected by the outcome of clinical trials [4–6]. Yet, due to the intricacies associated with transplantation procedures, expanded cells cannot be used directly for therapy. Thus, both short-term and long-term storage of expanded grafts is warranted for their convenient transportation and for their use in the future.

DMSO has been the most widely used cryoprotective agent for HSCs [7]. Reports from different studies indicate that the best cell recovery is obtained by controlled rate freezing with 5–10% DMSO. During cryopreservation, the change in temperature and osmolarity perturbs the membrane integrity and produces free oxygen radicals, which contribute to cell damage. Such cellular impairment experienced during freezing negatively affects the functionality of the cells [8,9].Thus, an optimal protocol for the cryopreservation of HSCs that could overcome freezing-induced damage needs to be developed to support HSC transplantation. Currently, various adaptations of freezing methods are being practiced which include the use of disaccharides as natural cryoprotectants and stabilizers for stem cell preservation[10–15].We have earlier demonstrated that the addition of certain bio-antioxidants to the conventional freezing medium improves post thaw recovery of human HSCs isolated from fetal liver and UCB [16,17].

HSCs and MSCs share a common niche *in vivo* and are known to have constant interactions with each other [18,19]. Therefore, MSCs have been extensively used as a scaffold for stromal support for *in vitro* expansion of HSCs. MSCs exert their effect on HSCs either via cell-cell contact or through diffusible molecules. Conditioned medium from MSCs (MSCs-CM) is rich in cytokines and various molecules like interleukins, growth factors, glycosaminoglycan and cell adhesion molecules[20,21].Thus, freezing of HSCs along with MSCs or the products derived from them represents a promising/effective strategy to preserve the quality of HSCs. In this study our aim was to evaluate the effect of MSCs-CM on the cryopreservation of UCB CD34^+^ cells that were expanded in suspension culture.

We report that CD34^+^ expanded cells, frozen with MSCs-CM, were better than the cells frozen in conventional medium alone in terms of viability, CD34^+^content and clonogenecity. We further show that the revived cells when cultured with MSCs-CM for an additional 48 hrs had improved their transplantation ability, as compared to those cultured without MSCs-CM. The cryo-protective effect of MSCs-CM was partly mediated by their anti-oxidative properties.CM of P-MSCs was shown to offer better cryoprotection than CM of C-MSCs. Thus, our data suggest that conditioned medium of MSCs can serve as a valuable constituent in freezing medium for the effective cryopreservation of expanded UCB cells.

## Materials and Methods

### Ethical approvals for Human samples and animal experiments

All protocols and methods for collection and processing of cord blood, cord tissue and placenta from local hospitals were approved by National Centre for Cell Science Ethics Committee (NCCS-IEC) and NCCS Committee for Stem Cell Research (NCCS-IC-SCR), which is in accordance with the Declaration of Helsinki. The format of consent forms and consenting procedures were also approved by the NCCS-IEC and NCCS-IC-SCR. All the samples were collected only after obtaining the written informed consent from the donors. The signed consent forms are stored as hard copies in the files.

The NOD/LtSZ-scid/scid mice (The Jackson laboratory, 610 St.Bar Harbor, Maine04609, USA) were bred in the animal facility of NCCS. The NOD/SCID mice were housed under aseptic conditions in individually ventilated caging systems (IVCs). Sterile cages were provided with autoclaved pellet diet, bedding and water which was changed at predetermined intervals. Per group (n = 5–10) 4–6 weeks old female mice were included. Protocols for animal experiments were approved and in accordance with IAEC-NCCS Institutional animal ethical committee-NCCS/CPCSEA- Committee for the Purpose of Control and Supervision of Experiments on Animals.(Approval number: IACUC-Institutional Animal Care and Use Committee, EAF-Experimental Animal Facility/2004/B-71(III)). Throughout the experiments animals were under the supervision of trained personnel and these mice were observed at least twice daily for signs of ill health like lethargy, decreased activity/mobility, physical attributes like hair coat and for evidence of diarrhea. The animals were sacrificed by CO_2_ gas asphyxiation which is considered as an established method that causes less suffering.

### Collection of Umbilical cord blood and isolation of CD34^+^ cells

Mononuclear cells were isolated by ficoll hypaque density gradient method(density 1.077 g/ml, Sigma Aldrich, St. Louis MO).In some experiments MNCs were directly used for freezing while for the expansion procedure CD34^+^ cells were isolated by positive selection method using Dynal beads as per manufacturer’s instructions (Dynabeads M-450 CD34; Dynal, ASA, Oslo, Norway).The purity of CD34^+^cells after isolation was checked by flow-cytomter as depicted in S1A Fig it was found to be 80–90%.The 10% contaminants are MNCs from cord blood. Since, these are differentiated cells they do not proliferate further and die off during the culture period leaving behind proliferative HSCs and progenitors or differentiated CD34^-^ cells. Moreover we have consistently used the same CD34^+^gating for all the experiments thus minimizing the probability of intra experimental variations.

### Isolation of MSCs and preparation of CM

MSCs were isolated from cord (n = 7) and placenta (n = 7) as described in reference [22]. Briefly, P-MSCs were isolated specifically from chorionic and decidual part of the placenta after removal of amniotic membrane and C-MSCs were obtained after removal of vitelline arteries and vein from Wharton’s jelly and the mesenchyme present within the different layers of cord which we isolate by squeezing the tissue mechanically. MSCs were grown till 80% confluency in RPMI1640(Sigma Aldrich,St. Louis MO) +20% FBS(Invitrogen, Grand Island, NY).Then, the monolayer was washed 3 times with PBS and plain RPMI1640(without FBS) was added to flasks. After 48hrs, CM was collected. Prior to use, CM was centrifuged at 200g for 15 mins to eliminate any cellular contamination.

The characterization of C and P-MSCs (representative samples) is depicted in S1B–S1D Fig.

### Ex vivo expansion and freezing of CD34^+^cells and UCB MNCs

5 x10^4^-10^5^ cells/well/500 μl were cultured in24 well plates with stem pro medium (GIBCO, Grand Island, NY), in the presence of Cytokines, IL-6, SCF, TPO, and Flt-3-L (all growth factors were from Peprotech Inc, Rocky Hill, NJ) at a final concentration of 25 ng/ml (standard expansion medium) for 10 days. In majority of our expansion experiments (95%) we had kept the seeding density as 10^5^ cells /well.At this seeding density there was no crowding even after 10 days of expansion thus the survival of cells was not hampered. Thus in most of our experiments we have used 10^5^ cells which is not only a widely used recommended seeding density but also validated in our earlier studies (22). But due to sample to sample variation and low CD34^+^ yield in some samples (5%) and in order to utilize the precious human samples, we had to reduce the seeding numbers to 5X10^4^ which is half of the standard 10^5^ cells. Equal number of cells /well were seeded in different sets of each experiment.

10^7^ MNCs and 10^6^ expanded CD34^+^were frozen either in Control (RPMI+20%FCS+10%DMSO) or C-MSCs-CM or P-MSCs-CM (50%(v/v)C-MSCs-CM/P-MSCs-CM + 50%(v/v)RPMI + 20%FCS+ 10%DMSO) with the cooling rate of -1°C /minute down to −80°C in a portable freezer (Freeze Control, Australia) and then located in liquid nitrogen. The cells were revived after 6–8 weeks of cryopreservation in liquid nitrogen. The cells were thawed by rapidly immersing the vials in a water bath at 37°C, and the cells were diluted with thawing medium (RPMI+ 20%BSA) and centrifuged at 4°C at 500g for 10 minutes. Cells were re-suspended in fresh medium and allowed to normalize at 37°C in 5% CO_2_for 2 hrs.

### Apoptosis detection by Annexin V/PI assay

10^5^ cells were washed with the PBS and re-suspended in 1X binding buffer (BD Biosciences, San Jose, USA). 5μL of Annexin V FITC (BD Biosciences, San Jose, USA) was added and incubated at room temperature for 20 minutes. Cells were washed to remove excess of Annexin V. PI was added at the concentration of 0.5μg/ tube and the samples were acquired on flow cytometer.

### Flowcytometry analysis

Cells were subjected to phenotypic characterization using panel of anti-bodies. For the detections of intracellular proteins the cells were permeablized using BD fixperm kit (BD pharmingen -San Jose, California) and the staining was done as per manufacturer’s instructions.The details of the antibodies used were as follows, CD34 APC/PE, CD45 PE/PE-CY-7 murine CD45.1 Pacific blue,CD33 PE/FITC,CD19 APC,CD3 FITC. (BD pharmingen- San Jose, California). Isotype matched antibodies were kept as controls. The fluorescently labeled cells were acquired on FACS Canto II and Aria (BD) and data was analyzed by FACS DIVA—version 5.0

### Colony-forming unit (CFU) assay

2x10^4^ cells were cultured in a semi-solid medium containing 1% methylcellulose (Sigma) with a combination of growth factors, SCF 20 ng/mL; GM-CSF 2 ng/mL; IL-3 4ng/mL; Epo 2U/mL.The plates were incubated at 37°C in 5% CO_2_ for 14 days.As per the differece in the morphology of the colonies they were scored as blast-forming unit erythroid (BFU-E), Colony Forming Unit-granulocyte-monocyte (CFU-GM), and Colony Forming Unit granulocyte-erythroid-monocyte-megakaryocyte (CFU-GEMM) and Colony Forming Unit-Megakaryocytes (CFU-MK).

### NOD/SCID Repopulation assay

The NOD/LtSZ-scid/scid mice (The Jackson laboratory, 610 St.Bar Harbor, Maine04609, USA) were bred in the animal facility of NCCS. The NOD/SCID mice were housed under aseptic conditions in individually ventilated caging systems (IVCs). Sterile cages were provided with autoclaved pellet diet, bedding and water which was changed at predetermined intervals.All the protocols were approved and in accordance with IAEC-NCCS Institutional animal ethical committee-National Centre for Cell ScienceNCCS /CPCSEA- Committee for the Purpose of Control and Supervision of Experiments on Animals.(Approval number: IACUC-Institutional Animal Care and Use Committee, EAF-Experimental Animal Facility/2004/B-71(III)). Throughtout the experiments animals were under the supervision of experts. Per group (n = 5–10) 4–6 weeks old female mice animals were included. 4–6 weeks old mice were sub lethally irradiated with 300 rads from a ^60^Co source (Gamma chamber 5000, BRIT, Navi Mumbai, India). 10^6^ revived cells were then infused introvenously into sub lethally irradiated mice. Throughout the experiments animals were under the supervision of trained personnel and these mice were observed at least twice daily for signs of ill health like lethargy, decreased activity/mobility, physical attributes like hair coat and for evidence of diarrhea. After 8 weeks, animals were sacrificed by CO2 gas asphyxiation which is considered as an established method that causes less suffering. Throughout Peripheral blood,bone marrow and spleen were harvested and engraftment was checked bydetecting human CD45^+^ cells against the murine CD45^+^ cells. The donor derived cells if found to be greater than 0.1% in total nucleated cells then it considered as successful engraftment. The following panel of antibodies were used: CD33 (myeloid cells), CD3, CD19 (lymphoid cells).The antibodies were purchased from BD pharmingen (BD; San Jose,California).The respective isotype antibodies (Beckton Dickinson; San Jose, California) were included as control. For staining of peripheral blood, 100 μl of blood was incubated with the anitbodies further RBCs were lysed with Lysis Buffer (BD,San Jose, California) and washed twice with PBS containing 0.1% BSA. A minimum of 1,00,000 events were acquired on FACS Canto II (BD, San Jose, California).

### Oxidative stress and ROS determination by DCFHDA

2x10^5^ cells/set were subjected to the 0.5mM H_2_O_2_ for 20 mins. The cells were then washed thrice with PBS and incubated further in either control or CM of C-/P-MSCs for 48 hrs. The pH,serum and glucose content and osmolality of test and control medium was checked and was found to be similar. After every 24 hrs cellular ROS was determined using DCFHDA dye (molecular probes, MD, USA) as per the manufacture’s instruction. Briefly the cells were washed with PBS and stained with 5uM DCFHDA for 20 mins at ambient temperature. The cells were washed twice with PBS and sample was acquired using FACS Canto II (BD,San jose,USA).

### Isolation of exosomes and exosome free supernatant

MSCc-CM was centrifuged initially at 5000 rpm for 20 mins.The supernatant after this centrifugation step was subjected to one more round of centrifugation at 40000 rpm for 90 mins at 4°C.The supernatant was then used as exosome free fraction and the exosomes(pellet) were resuspended in the fresh medium and used for the experiments.

### Realtime PCR

Revived cells were cultured with standard expansion medium or MSCs-CM, were cultured for additional 48 hrs. 5x10^5^ cells were used for mRNA isolation using mRNA direct kit(DYNAL, invitrogen) as per manufacture’s instruction.RNA quantification was done using Nanodrop spectrophotometer (ND1000). cDNA synthesis was carried out by using ‘high capacity cDNA archive kit’ (Applied Biosystems, Foster City, CA). 1 μg of cDNA was used for real time PCR using the SYBR-Green PCR master mix (Applied Biosystems, Foster City, CA) and with 7500 ABI-prism sequence detection system (Applied Biosystems). Negative controls were comprised of samples without reverse transcription step. Results obtained were normalized to GAPDH as housekeeping gene. Following is the list of primers used (Table 1).

**Table 1:** List of primers.

Gene	Forward(5’-3’)	Reverse(5’-3’)
Human GAPDH	ACTGCCACCCAGAAGACTGT	CCATGCCAGTGAGCTTCC
Human GPX-1	CCCAACTTCATGCTCTTCGA	ATGTCAATGGTCTGGAAGCG
Human Catalase	TGTGAACGTGAATGAGG	GATTTGCCTTCTCCCTTGCC
Human SOD-1	GTGAAGGTGTGGGGAAGCAT	CCCAAGTCTCCAACATGCCT

### Statistical analysis

The statistical differences between groups were analysed by one way repeated measure analysis of variance (One WAY RM ANOVA)using the SIGMA STATsoftware(Jandel Scientific Corporation,San Rafael, CA) for all the experiments.Each experiment was done in triplicates with the repetition on 3–5 cord blood unit.(N = 3–5)The values were plotted as mean ± standard deviation Probability value: *p≤0.05,**p≤0.01, & ***p≤0.001 were considered statistically significant.

## Results

### Cryopreservation of expanded UCB 34^+^ cells with MSCs-CM improves recovery, survival and functionality of the revived cells

**Fig 1:**
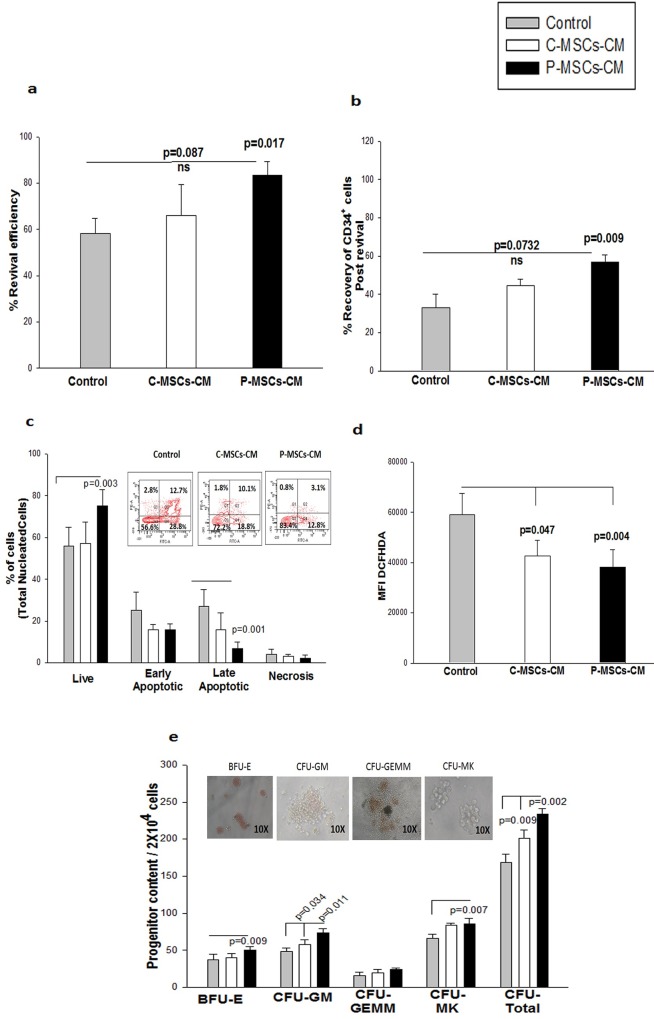
Improved recovery of expanded 34^+^ cells after freezing with MSCs-CM. Expanded cells were subjected to the cryopreservation in either control medium or C-MSC-CM or P-MSCs-CM. (A) Revival efficiency was calculated by trypan blue dye exclusion method. Significantly higher efficiency was obtained for the cells frozen with P-MSCs-CM.(B)P-MSCS-CM displayed significantly recovery of the CD34^+^ cells as compared to control.(C) Annexin V/PI staining on Total nucleated cells showed significant increase in the viable cells and reduction in the late apoptotic population in the cells frozen with P-MSC-CM. Inset depicts the FACS dot plots for representative samples(D)CD34^+^cells frozen MSCs-CM had significantly lower level of total cellular ROS.(E) The cells frozen in P-MSCs-CM displayed higher blast-forming unit erythroid (BFU-E), granulocyte -monocyte(GM),)and Megakaryocytes (MK) colonies and total colonies when compared to C-MSCs- CM and control set. Inset depicts the representation of each colony type. Data is represented as Mean ± standard deviation from 5 different independent experimental sets. (ns-non significant)

Freshly isolated UCB CD34^+^ cells were expanded in standard expansion medium (Exp-Medium) for 10 days and were then subjected to rate controlled freezing without (control) or with conditioned medium of C-MSCs (C-MSCs CM set) or P-MSCs (P-MSCs CM set).The frozen cells were revived after 6–8 weeks, the viable cells were counted by trypan blue dye exclusion method and revival efficiency was estimated. Cells frozen with C-MSCs-CM (65.2±3.43%) and P-MSCs CM (82.54±1.5%) displayed a higher revival efficiency as compared to those in the control set (59.7±1.42%) (Fig 1A).This difference was significant in the P-MSCs-CM set. The percentage of CD34^+^ cells in the expanded unit detected prior to their cryopreservation was considered as 100% against which the recovery of CD34^+^ cells post revival was calculated. The P-MSCs-CM set showed significantly higher recovery of CD34^+^ cells as compared to the control set. Though the C-MSCs–CM set also displayed higher recovery of CD34^+^cells but didn’t reach significance (Fig 1B). We have consistently used the same CD34^+^ gating for all the experiments thus minimizing the intra experimental variations

Next, we subjected the revived cells to PI staining and the viability was analyzed by flow-cytometry. Expanded CD34^+^cells frozen in control medium had a significantly higher percentage of dead cells than those frozen with C-MSCs-CM and P-MSCs-CM set (S2A Fig). S2B Fig depicts the FACS profile of the representative sample. We then determined the actual percentage of cells undergoing apoptosis by performing Annexin V/PI staining on the revived cells. The percentage of viable total nucleated cells was found to be significantly higher in the P-MSCs-CM set than the control set. The viable cells in C-MSCs–CM set were comparable to the control set. A significant reduction in the late apoptotic population was also observed in cells frozen with P-MSCs-CM as compared to control (Fig 1C). The Inset depicts a representative FACS profile showing the reduction in the apoptosis in TNC in MSCs-CM set. Though there was reduction in apoptosis in both CM sets the difference was significant only in the P-MSCs-CM set. In the gated CD34^+^ cells, the viability was significantly higher in P-MSCs-CM set as compared to the control(S2C Fig). The representative FACS profiles depicting a reduction in the apoptosis in CD34^+^ cells in the MSC-CM sets is shown in S2D Fig. We then assessed the levels of ROS in all the three sets and a significant reduction was observed in the intensity of DCFHDA in both MSCs-CM sets as compared to control. (Fig 1D).Further, to check the functionality of the frozen cells, revived cells were then subjected to the *in vitro* methyl cellulose based colony formation assay. The P-MSCs-CM and C-MSCs-CM sets had significantly higher progenitors CFU-GM and CFU-total, as compared to the control set. Additionally, in the P-MSCs-CM set a significantly higher content of BFU-E and CFU-MK was observed than in the control set. (Fig 1E).The representative phase contrast images of each colony type is shown as an inset.

Our observations are suggestive of a cryoprotective effect of MSCs -CM on the expanded frozen CD34^+^ cells.

### Augmentation in proliferation and functionality of the expanded cells cryopreserved and primed with MSCs-CM

**Fig 2:**
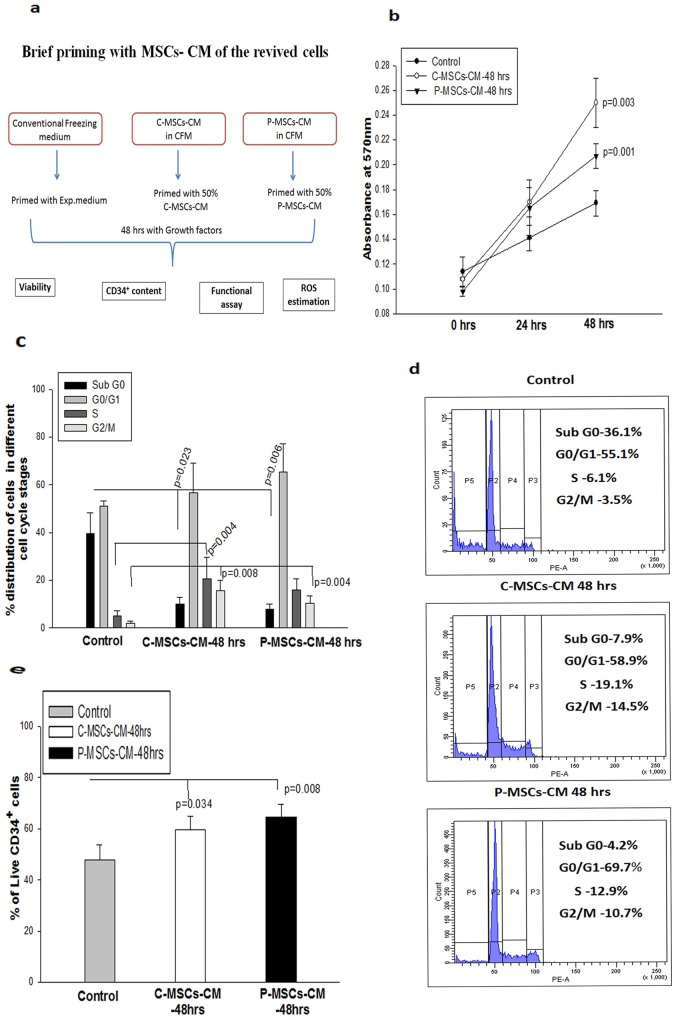
Brief priming of the revived cells with MSC-CM improves their proliferation and survival. Cells frozen with CM and primed for 48 hrs with MSCs-CM (A) Flow chart depicting the experimental design.(B)Higher proliferation was seen in the cells primed with MSCs-CM.(C)The cell cycle analysis shows drastic reduction in the sub G0 phase with an increase in the percentage of cells in the S and G2/M phase in MSCs-CM set. (D)The histogram representation demonstrating the distribution of the cells in various stages of cell cycle. (E)Higher percentages of live CD34^+^ cells were found in the MSCs-CM as opposed to control. Data is represented as Mean ± standard deviation from 3 different independent experimental sets.

The cryopreserved cells from all 3 sets were revived and were cultured for 48 hrs. in standard expansion medium (Exp-Medium)to assess freezing-induced defects. The experimental design is depicted in S3A Fig. We observed only a marginal increase in their proliferation (S3B Fig), cell cycle analysis (S3C Fig) and viability of CD34^+^ cells (S3D Fig) in the cells frozen with MSCs-CM as compared to those frozen in control medium. The sub G0 population representing the apoptotic fraction was however significantly reduced in MSCs-CM. Similarly, the cells frozen with control medium and exposed to MSC-CM post-revival exhibited a moderate increase in the proliferation and CD34^+^ content (Data not shown). In order to further improve the quality of the cells frozen with CM, we incubated the revived cells with the Exp-medium containing 50% (v/v) of C-MSCs-CM(C-MSCs-CM-48hrs) or 50% (v/v)of P-MSCs-CM (P-MSCs-CM 48hrs)or 50% (v/v plain RPMI-control)for 48hrs.The flow diagram of experimental design is depicted as Fig 2A. Statistical comparison was done between the cells which were frozen and primed without MSCs-CM (Control) vs the cells frozen and primed with MSCs–CM(Test).C-MSCs-CM-48hrs or P-MSCs-CM-48hrs stimulated the proliferation of the cells as is evident from data from the MTT assay, as compared to the cells frozen with control medium and grown in the Exp-Medium. (Fig 2B).Since we observed superior maintenance of revived CD34^+^ cells that were frozen with MSCs-CM and cultured for 48 hrs in MSCs-CM as compared to cells frozen and cultured without CM, all our further experiments were restricted to the following 3 sets i.e

Control-Cells frozen without CM and cultured without CM (control),

C-MSCs-CM-48hrs -Cells frozen with C-MSCs-CM and cultured with C-MSCs-CM,

P-MSCs-CM-48hrs—Cells frozen with P-MSCs CM and cultured with P-MSCs-CM

The cell cycle analysis displayed a significant reduction in the sub G0 phase and increase in the G2/M phase in the cells frozen with CM and continually grown in CM, as compared to those in the control. C-MSCs-CM also showed a significant increase in the S phase (Fig 2C). Fig 2D depicts a histogram, representing the distribution of the cells in different stages of the cell cycle in the 3 sets. Thus, the scattering of the revived cells in various stages of the cell cycle co-related well with the data of the proliferation assay.We then determined the percentage of viable CD34^+^ cells in the cultured sets. Both the CM sets harbored significantly higher live CD34^+^ cells (59.60 ± 9.16% and 64.30 ± 18.16% respectively)as compared to those in the control sets (46.73 ± 10.19%) Fig 2E. These observations indicate that the conditioned medium of MSCs provides superior cryo-protection, which can be ameliorated by the addition of CM during culture.

**Fig 3:**
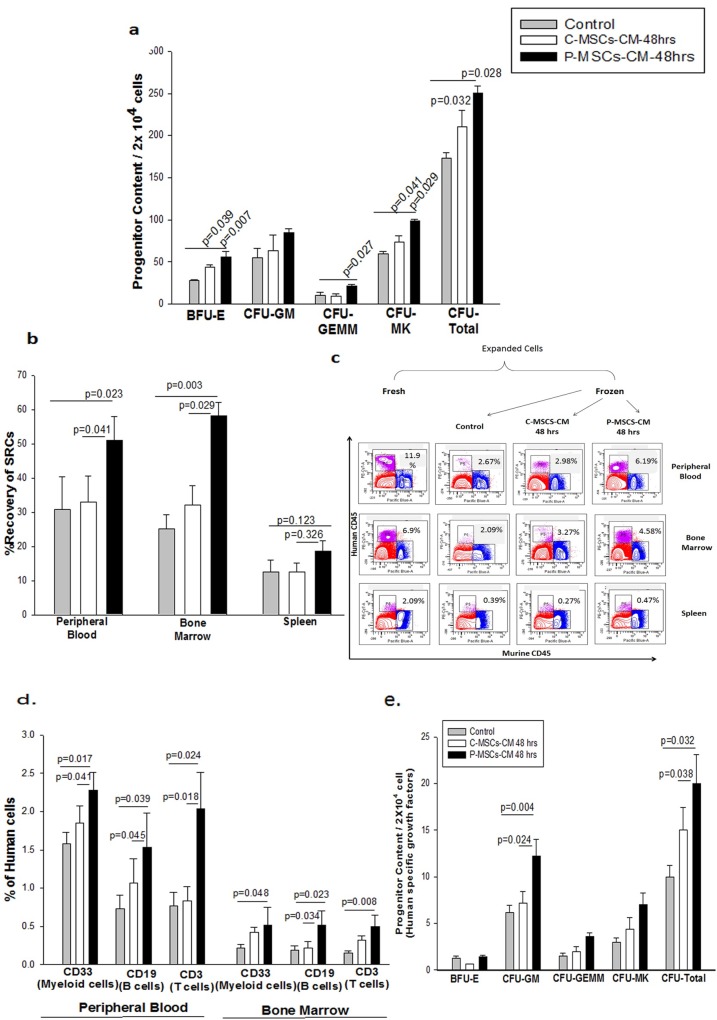
Brief priming of the revived cells which were frozen with MSCs-CM with MSC-CM improves *in vitro* and *in vivo* functionality of the revived cells. (A)Significantly higher progenitor content was found in the MSCs-CM sets as estimated by *in vitro* colony formation assay. The cells frozen in P-MSCs-CM displayed higher blast-forming unit erythroid (BFU-E), granulocyte-erythroid-monocyte-megakaryocyte (GEMM),)and Megakaryocytes (MK) colonies and total colonies when compared to C-MSCs- CM and control set(B) The cells primed with P-MSCs-CM displayed significantly higher recovery of SRC human chimerism (%Human CD45^+^cells) in peripheral blood and bone marrow signifying superior engraftment potential than C-MSCs-CM and control set after 8 weeks of transplantation when compared with their freshly expanded counterparts.(C). The engrafted cells were able to generate the multi-linage differentiation as estimated by detection of human derived CD33 (myeloid cells), CD19 (B-Cells) and CD3 (T -cells) in the peripheral blood and bone marrow.(D)To further reconfirm the origin of the cells we performed CFU assay using human specific growth factors. We observed significant increase in the CFU-GM and CFU-total in the set which was primed with P-MSCs-CM in comparison to control and C-MSCs-CM set. Data is represented as Mean ± standard deviation from 3 different independent experimental sets which had n = 5–10 mice/ group.

We assessed the functionality of the frozen and re-cultured cells by CFU assay. A significantly higher clonogenecity (total colonies) was obtained from the cells frozen and re-cultured in C-MSCs-CM set as well as P-MSCs-CM set in comparison to the control set (Fig 3A).P-MSCS-CM supported significantly higher BFU-E, CFU-GEMM,CFU-MK as compared to control. C-MSCS-CM resulted in a significant increase in BFU-E and CFU-MK (Fig 3A).

We next investigated the SCID repopulation capability (SRC) of revived cells which were primed with MSCs-CM post-revival. Freshly expanded CD34^+^ cells were considered as positive control to calculate the recovery of SRC of the expanded, frozen cells that were primed with/ without MSCs-CM. We calculated the recovery of SRC by comparing the level of engraftment attained with the cells cryopreserved and primed with MSCS-CM with that of fresh *ex vivo* expanded (unfrozen) CD34^+^ cells.10^6^ cells from the three sets were infused into sub-lethally irradiated NOD/SCID mice. The three experimental sets were similar to our earlier experiments.The statistical comparison was made between the frozen revived sets i.e. control (primed without CM) vs test (primed with CM) sets.

The presence of human cells was detected in the peripheral blood, bone marrow and spleen of the mice 8 weeks post-infusion. A significantly higher recovery of human CD45^+^ cells was obtained in the peripheral blood (1.7 fold) and bone marrow (2.4 fold) of mice which received cells from P-MSCs-CM set, as compared to the control. We observed no significant difference between the engraftment provided by the control and the cells from C-MSCs-CM set in peripheral blood and bone marrow (Fig 3B). We also observed no difference in the human chimerism in the spleen of mice between all the three sets. The representation FACS dot plots depicting the human CD45^+^ cells probed against the murine CD45^+^ cells in the peripheral blood, bone marrow and spleen of mice(Fig 3C).Further we checked if the engrafted cells were able to differentiate into myeloid and lymphoid lineages which were probed in the peripheral blood and bone marrow of the mice. A significantly higher percentage of CD 33, CD19 and CD 3 cells of human origin was observed in the peripheral blood and bone marrow for the mice infused with cells from P-MSCs-CM set (Fig 3D). The difference between C and P-MSCs–CM sets were also significant, underscoring superiority of the engraftment potential of P-MSCs-CM set over C-MSCs-CM set. We further verified the origin of the progenitors by performing CFU assay using human specific growth factors. P-MSCs-CM showed a significantly higher CFU-GM and CFU-total content as compared to the control as well as C-MSCs-CM set (Fig 3E). Thus, it can be concluded that the revived CD34^+^ cells re-stimulated with P-MSCs-CM displayed better multi-lineage engraftment in NOD/SCID mice as compared to the other two sets.

### CM of MSCs provides cryoprotection by reducing the oxidative stress in the cryopreserved cells

**Fig 4:**
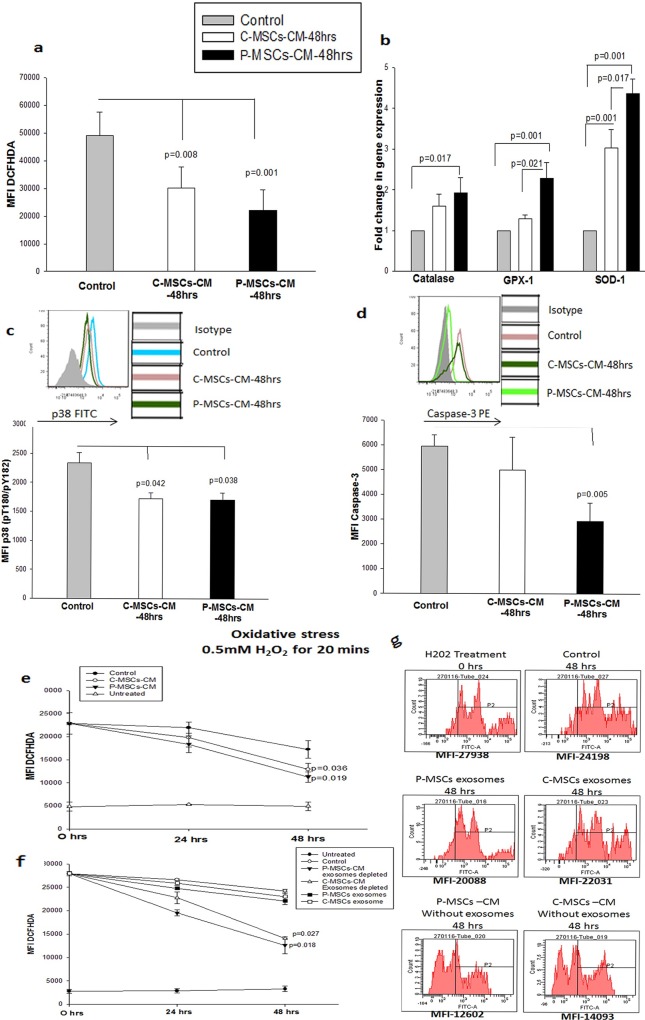
MSCs-CM provide cryo-protection by reducing oxidative stress. (A)Priming of revived cells with MSCs-CM significantly reduced the level of cellular ROS.(B)The reduction seen in the MSCs-CM set was due to associated increase in the mRNA levels of anti-oxidant enzymes,Catalase,GPX-1 and SOD-1 in these sets as estimated by real time PCR.(C) On the background of lower ROS levels the level of phosphorylated p38(pT180/pY182) was found to be significantly lower in MSCs-CM sets. (D) As a consequence of activation of p38 we checked the levels of caspase-3 in the three sets. P-MSCs-CM set displayed a significant reduction in the Caspase-3 level. (E) To further validate the anti-oxidant capacity of MSCs-CM, we subjected freshly isolated CD34^+^ cells to oxidative stress (0.5mM H_2_O_2_ for 20 mins). Significant decrease in the ROS levels was seen in the sets cultured with MSCs-CM as opposed to stressed control set. (F) We fractionated MSCs-CM into exosomes and exosome free supernatant and incubated the cells treated with H2O2 with these two fraction for 48 hrs. Drastic reduction in the ROS levels were seen in the exosome free supernatant of MSCs-CM. (G) The representative FACS histogram depicting the reduction in intensity of DCFHDA in the exosome free supernatant fraction. Data is represented as Mean ± standard deviation from 3 different independent experimental sets.

To decipher the mechanism underlying the cryoprotective effect of CM of MSCs we evaluated oxidative stress in the cryopreserved and re-cultured CD34^+^ cells. A drastic reduction was observed in the total cellular ROS in the cells primed with the C-MSCs-CM(MFI-1.6 fold) and P-MSCs CM(MFI –2.2 fold) as compared to the control set as estimated by DCFHDA staining (Fig 4A). We then, evaluated mRNA levels of anti-oxidant enzymes viz, Catalase, Glutathione peroxidase-1 and Superoxide dismutase-1 in the CD34^+^ cells cryopreserved and re-cultured in the MSCs-CM by real time PCR. The C-MSCs–CM set displayed a significant increase in mRNA levels of GPX-1 and SOD-1 as compared to the control set. On the other hand the P-MSCs-CM set displayed a significant upregulation of catalase, GPX-1 and SOD-1, as compared to control (Fig 4B). Thus, it can be concluded that incubation of revived cells with CM of MSCs rescues the cells from oxidative stress incurred during cryopreservation.

To further confirm the reduction in the cellular oxidative stress, we checked the levels of phosphorylation of p38 at pT180/pY182, which is the downstream target of ROS accumulation in the cell. We observed no difference in the percentage of CD34^+^p38^+^ cells in all the sets (data not shown) but a significant 1.3 fold reduction was observed in the MFI of p38 for both the CM sets in comparison with control(Fig 4C.)The inset depicts a representative FACS overlay displaying the lower MFI values for CM of MSCs in comparison with control. We then assessed the level of caspase-3, a target of p38 phosphorylation, in the cells primed with CM of MSCs. Cells frozen and re-cultured with P-MSCs CM had significantly lower levels of caspase-3 (2 fold) as compared to control cells. The reduction of Caspase-3 in the C-MSCs-CM set was not statistically significant. (Fig 4D). The inset depicts the overlay showing the reduced expression of Caspase-3 in the CM-P-MSCs set.

### MSCs CM also reduced oxidative stress induced by H_2_O_2_

To further validate our observation that MSCs-CM possess anti-oxidant properties, we subjected the freshly isolated CD34^+^ cells from UCB to the oxidative stress by incubating the cells with 0.5mM H_2_O_2_ for 20 mins. These cells were then incubated for a period of 48 hrs. as three sets shown below:

Exp-medium- CD34^+^ cells treated with H_2_O_2_ incubated with control medium (without CM)

C-MSCs-CM-48 hrs- CD34^+^ cells treated with H_2_O_2_ incubated with C-MSCs-CM

P-MSCs-CM-48 hrs -CD34^+^ cells treated with H_2_O_2_ incubated with C-MSCs-CM

Negative Control- Untreated cells incubated with control medium (without CM)

The kinetics of ROS levels in the 3 sets was followed for 48 hrs. We observed a lower level of DCFHDA in the cells incubated with CM of MSCs and this difference reached significance at 48 hrs. (Fig 4E). To further determine if the antioxidant capacity was due to exosomes secreted by MSCs, we performed a similar experiment with the MSCS-CM fractionated into exosomes and exosome- free supernatant. We observed a significant reduction in the ROS levels when the stressed cells were incubated with exosome free supernatant of MSCs-CM (Fig 4F).We observed a marginal reduction in the exosome fraction also but this reduction was not significant. The representative histogram depicting the mean fluorescence intensity is shown in the Fig 4G. These data directly supports the observation that anti-oxidant activity of MSCs–CM resides in the exosome free supernatant.

### P-MSC-CM is closer to catalase in antioxidant activity

In another experiment we used catalase as an additive to conventional freezing medium for cryopreservation of ex vivo expanded cells. This set served as a positive control to quantify the anti-oxidant potential of CM of C-and P-MSCs. The cells cryopreserved with catalase demonstrated maximum cryoprotection with respect to cell yield, total CD34^+^ cells and clonogencity after revival as compared to control and CM of C-MSCs. (S4A, S4B and S4C Fig). However no statistically significant difference was observed between the cryoprotection offered to CD34 cells by 100ug/ml of catalase and CM of P-MSCs. Considering the activity of catalase as 100%, CM of C and P-MSCs offered approximately 68% and 78% respectively of antioxidant capacity. We also evaluated the cellular ROS levels in the revived cells and observed significantly lower DCFHDA intensity in the cells cryopreserved with catalase, C-MSCs-CM and P-MSCs-CM than in the control (S4D Fig).

### Cryoprotective effect of CM of MSCs was not only restricted to CD34^+^ cells but also could be extended to CB MNCs

**Fig 5:**
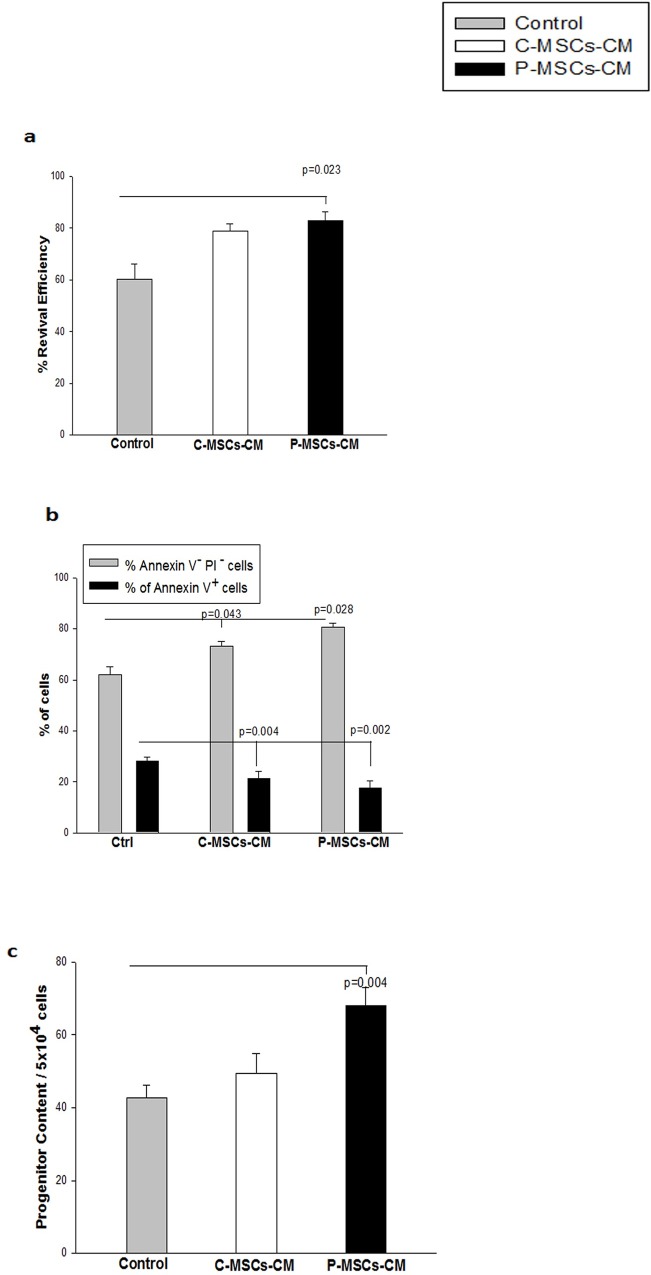
MSCs-CM provide cryo-protection to CB MNCs. UCB mononuclear cells (MNCs) were subjected to cryopreservation with Conventional freezing medium and with MSCS-CMs. The revival efficiency.viability progenitor content was assessed immediately after revival. (A) Significantly higher revival efficiency was obtained with P-MSCs-CM set.(B) Freezing of UCB MNCs with MSCs-CM yielded higher percentage of live(Annexin V^-^ PI−cells)with significant reduction is seen in the dead cells(Annexin V^+^ cells).(C)UCB MNCs harbored superior progenitor content when frozen with P-MSCs-CM as compared to control set. Data is represented as Mean ± standard deviation from 3 different independent experimental sets.

To determine whether the MSC-s CM provide cryoprotection to UCB Mononuclear cells (MNCs),CB MNCs were frozen with MSCs-CM. MNCs frozen with P-MSCs-CM had significantly higher revival efficiency as compared to cells frozen without CM (Fig 5A).The difference between C-MSCs–CM and control was not statistically significant. AnnexinV/PI analysis of cryopreserved cells further displayed that MNCS frozen with C-MSCs-CM (73.2±2.82%) and P-MSCs CM (80.66±1.38%) showed a corresponding increase in the number of viable cells (AnnexinV^–^PI^-^) over the Control (62.03±3.18%),along with a corresponding decrease in the levels of apoptosis (Annexin V^+^) (Fig 5B). We further carried out CFU assay to ensure the functionality of the cryopreserved cells and observed better retention of progenitors in the presence of CM of P-MSCs than with the control medium used for cryopreservation (Fig 5C).

This data suggests that P-MSCs-CM was better than C-MSCs–CM in offering cryoprotection to the CB MNCs.

## Discussion

A global increase in cord blood banks has supported the utility of UCB in HSC transplantations [1–3].The *ex vivo* expansion of HSCs enable the production of sufficient numbers of cells for transplantation from even the smallest cord blood unit stored in bank [4–6]. One of the approaches to increase the utility of the expanded HSCs is their successful cryopreservation [23].

In our earlier studies, we observed that cells expanded in co-cultures with MSCs as feeders were more cryo-resistant than those expanded in suspension culture (unpublished data). The MSCs are known to interact with HSCs either through cytokines secreted by them or by cell- to- cell adhesion [18]. But MSCs interactions with HSCs are not possible at sub-zero temperatures. Therefore, the role of cytokines secreted by MSCs would assume importance in the cryoprotection of CD34^+^ cells. MSCs are known to secrete a variety of pro-survival factors in cardiovascular and neurodegenerative diseases, reducing apoptosis, inflammation and oxidative stress in these conditions [24–27]. Thus, we sought to investigate if MSCs-CM would similarly exhibit a cryo-protective effect on the frozen HSCs.

Lin et al [28]have reported that CM from Wharton’s jelly derived MSCs provides signals to the CD34^+^ cells thus supporting better survival than conventional freezing medium(CFM). In our studies, though the C-MSCs-CM was found to be a better cryoprotectant than CFM the difference was not as striking as reported by Lin et al. These differences in our findings may be due to two main reasons. First, the CM used by Lin *et al*. was collected after 24 hrs as opposed to the 48 hrs in our experiments. We did not observe any significant cryoprotective activity in 24 hrs. CM thus included 48 hrs CM in our study (Data not shown). Second, Lin *et al*. proposed that exosome mediated transfer of microRNA was involved in cryoprotection of the cells. On the contrary, fractionation of conditioned medium in our studies revealed that exosome free supernatant was more efficient at protecting the cryopreserved cells. Lin et al. study lacked in in *vivo* transplantation assay in NOD/SCID mice, while we performed gold standard NOD/SCID transplantation assay to reinforce our claim.

According to our earlier findings [22], we observed that P-MSCs-CM had superior cryoprotective activity than C-MSCs-CM. This difference may be attributed to the distinct secretory profiles exhibited by C and P-MSCs as described in this report [22]. Although, P and C-MSCs are in close anatomical proximity, both exhibited differences in their characteristics, such as higher concentration of HGF in P-MSCs- CM compared to C-MSCs-CM [22]. HGF is known to have anti-oxidant effect [20,21] which might have contributed to the superior cryoprotection provided by the P-MSCs-CM. Therefore, the observed differences are most likely to be due to a variation in the contents of the CM rather than any change in the protocol during CM preparation.

The viable count estimated immediately after the revival was significantly higher than that observed just before infusion as demonstrated by Duchez et al and Kudo et al [29,30]. This decrease in the cell counts was linked to the initiation of the apoptotic cascade. A priming period of 48 hrs was also shown to be essential by Giarratana et al [31] to evaluate the quality of expanded graft prior to its infusion into patient.In addition, such priming of the cells after revival also leads to the dilution of DMSO and FBS used during freezing, thus avoiding adversities associated with these two components. Therefore, it would be desirable to incubate revived cells for 48 hrs. to ensure the better rescue of the cells from cryo- injuries. In our culture system, we observed marginal increase in the proliferation when revived cells were cultured with Exp-medium but after inclusion of 50%of MSCs-CM in the medium cells primed for 48 hrs. in CM showed improvement in proliferation and functionality.

ROS ^low^ HSCs have a higher engraftment potential [32–36].Thus, the observed superior SRC potential of the CD34^+^ cells cryopreserved and primed with MSCs-CM in our test sets could be attributed to the lower levels of ROS in these cells. These observations further validated when we subjected freshly isolated CD34^+^ cells to oxidative stress with H_2_O_2_. To rule out the possibility that oxidative stress may be due to a change in the osmolality, pH, glucose and serum conditions etc. we treated freshly isolated CD34 ^+^ cells with a known inducer of oxidative stress i.e. H_2_0_2._In other words the culture media in the treated and untreated cells was kept constant to ensure that oxidative stress was specifically due to H_2_0_2_ treatment. Thus, as we have used the same medium for treated and untreated cells it rules out the possibility of any change in the osmolality, pH, glucose and serum conditions and clearly indicates that MSCs-CM have a strong anti-oxidant ability. Upon further investigation about whether the anti-oxidant effects exerted by MSCs-CM were due to exosomes or the exosome -free supernatant. The majority of the anti-oxidant capacity was observed in the exosome free supernatant and not in the exosomes. Since, we did not see any anti-oxidant effect when we had added the exosomes isolated from the similar volumes of conditioned medium.(Concentrated exosome culture), we did not perform any further serial dilutions of exosomes as it seems very irrelevant. This observation is consistent with the studies reported by Kim et al.[37] where they found anti-oxidant properties associated with cytokines (proteins) like HGF,IGFBPs,PGE-2 etc. secreted by the MSCs.

Higher ROS levels leads to activation of the p38 MAPK pathway, resulting in higher levels of phosphorylated p38 [32]. We observed a significant down-regulation of p38 phosphorylation and hence caspase-3 activation [38–40] in the cells frozen and re-cultured with MSCs-CM further confirming lower levels of ROS in them. Based on the inverse co-relation between levels Bcl-2 and caspase-3 as reported in [41],we speculated that the drastic reduction in caspase -3 expressions in the P-MSCs-CM as compared to C-MSCs-CM could be due to high endogenous levels of Bcl-2 (data not shown) which probably resisted initiation of apoptosis in P-MSCs-CM set.

To determine the applicability of MSCs-CM in the cryopreservation of other cells, we tested its effect on CB MNCs, which are easiest to isolate form UCB than CD34^+^ cells. The MNCs frozen with MSCs-CM displayed a remarkably improved recovery of TNC, survival and functionality upon revival. These data also suggest that CB banks could use MSCs-CM to freeze MNCs, revive them and expand the CD34^+^ cells with CM before transplantation in patients. While we acknowledge the variability in the secretory profiles of MSCs from different donors, the consistency of results observed in 7 independent MSCs samples in our studies is rather encouraging. We further propose to make a detailed analysis of the conditioned medium, which would help in formulating a more defined medium for freezing HSCs. It is also relevant to assess the correlation between the time stored and survival benefits to the cryopreserved cells but we did not check this aspect in present study as it is beyond the scope of the current manuscript. But, in our earlier study [16] we observed that cryoprotective effect of membrane stabilizer and anti-oxidant could be maintained up to 1.5 years. So, we believe that MSCs-CM can similarly be effective for longer periods of time. However this needs to be validated. Further, others and our earlier report [42, 43] emphasized that the FBS in the freezing medium can be efficiently replaced by the autologous plasma/serum for the freezing of MSCs and HSCs. MSCs-CM represents an economical substitute for chemically-synthesized freezing medium containing growth factors and will have a broader impact in the field of transfusion medicine.

## Conclusion

Ex vivo expansion and their effective cryopreservation represent a valuable step in increasing the clinical applications of UCB. We here report that conditioned medium from MSCs provide better cryoprotection than the conventional freezing medium. We report that MSCs-CM reduce the oxidative stress in the ex vivo expanded UCB CD34^+^ cells during cryopreservation, reducing the level of apoptosis in them. This protective effect of MSCs-CM was found in the exosome free supernatant fraction and was not mediated by exosomes secreted by them. Taken together, our data strongly recommends the use of MSCs-CM as a valuable constituent in the freezing medium and for priming medium for the effective cryopreservation of CD34^+^ cells as well as CB MNCs.

### Endnote

A part of this data was presented by Darshana Kadekar at the 44^th^ Annual Scientific meeting of ISEH-International Society for Experimental Hematology at Kyoto in September 2015.

## Supporting Information

S1 Fig Well characterized CD34^+^ cells and MSCs were used for all the experiments. Isolated primary cells were subjected to phenotypic and functional characterization to check their purity prior to the experiment.(A)Representative histogram for 3 cord blood units to check their Purity after isolation from MNCs.(B) Fibroblastic morphology exhibited by C and P-MSCs as seen under phase contrast microscope(10X).(C) Overlays of histogram of representative samples of C and P-MSCs exhibiting expression of markers like CD44, CD90, CD73, CD105 and with no expression of CD45,CD34,CD14,CD19,CD11b and HLA-DR.(D) Upper panel represents osteogenic differentiation of MSCs by staining with alizarin red S. Middle panel is for adipogenic differentiation confirmed after lipid droplets stained by oil red o. Chondrogenic differentiation of C and P-MSCs as pellets confirmed with H&E staining.
(TIF)

S2 Fig Reduced levels of apoptosis was observed in the cells frozen with MSCs-CM. Expanded cells were subjected to Propidium iodide assay to check for the membrane damage and thus the viability.(A)The cells frozen with MSCs-CM had significantly lesser number of PI^+^ cells as compared to control.(B)The FACS profile of the representative samples depicting reduction in the percentage of PI^+^ cells in MSC-CM in comparison to control. (C) The level of apoptosis in the gated CD34^+^ cells was found to be significantly reduced in the P-MSCs-CM set. The % of viable cells was also higher p-MSCs-CM set. (D) FACS profile of representative sample depicting the distribution of revived CD34^+^cells at the various stages of apoptosis. Data is represented as Mean ± standard deviation from 3 different independent experimental sets.
(TIF)

S3 Fig Re-culturing of revived cells with Standard expansion medium had no effect on their recovery. (A) Flow chart depicting the experimental design. (B)No significant difference in the proliferation was observed in the cells frozen in control or MSCs-CM and then re-cultured in Exp.medium. (C)The cell cycle analysis of these cells shows drastic reduction in the sub G0 phase with no change in the percentage of cells in the S and G2/M phase.(D)No difference was observed in the viability of CD34^+^ cells in all the three sets. Data is represented as Mean ± standard deviation from 3 different independent experimental sets.
(TIF)

S4 Fig MSCs-CM displayed similar anti-oxidant capacity as that of 100μg/ml of catalase. The expanded cells were frozen and primed with Control, Catalase (100μg/ml) as an additive in the CFM, C-MSCs-CM and P-MSCs-CM.(A) The cells frozen and re-cultured with catalase displayed maximum cell yield of total nucleated cells. The increase was also significant in the P-MSCs-CM set. (B)The CD34^+^ cells were also higher in catalase and P-MSCs-CM set.(C)Freezing and priming of expanded CD34^+^ cells with catalase resulted in to augmented clonogenecity of these cells. MSCs-CM set also displayed higher yield of blast-forming unit erythroid (BFU-E), granulocyte -monocyte(GM), granulocyte-erythroid-monocyte-megakaryocyte (GEMM)and Megakaryocytes (MK) colonies (d)Drastic reduction in total cellular ROS was seen in all the three sets as opposed to control set. Data is represented as Mean ± standard deviation from 3 different independent experimental sets.
(TIF)
