# Supplementary figures and images for: Conditioned Medium from Placental Mesenchymal Stem Cells Reduces Oxidative Stress during the Cryopreservation of Ex Vivo Expanded Umbilical Cord Blood Cells
Source: PLoS One. 2016 Oct 25;11(10):e0165466. doi: 10.1371/journal.pone.0165466 (PMC5079553; doi:10.1371/journal.pone.0165466)

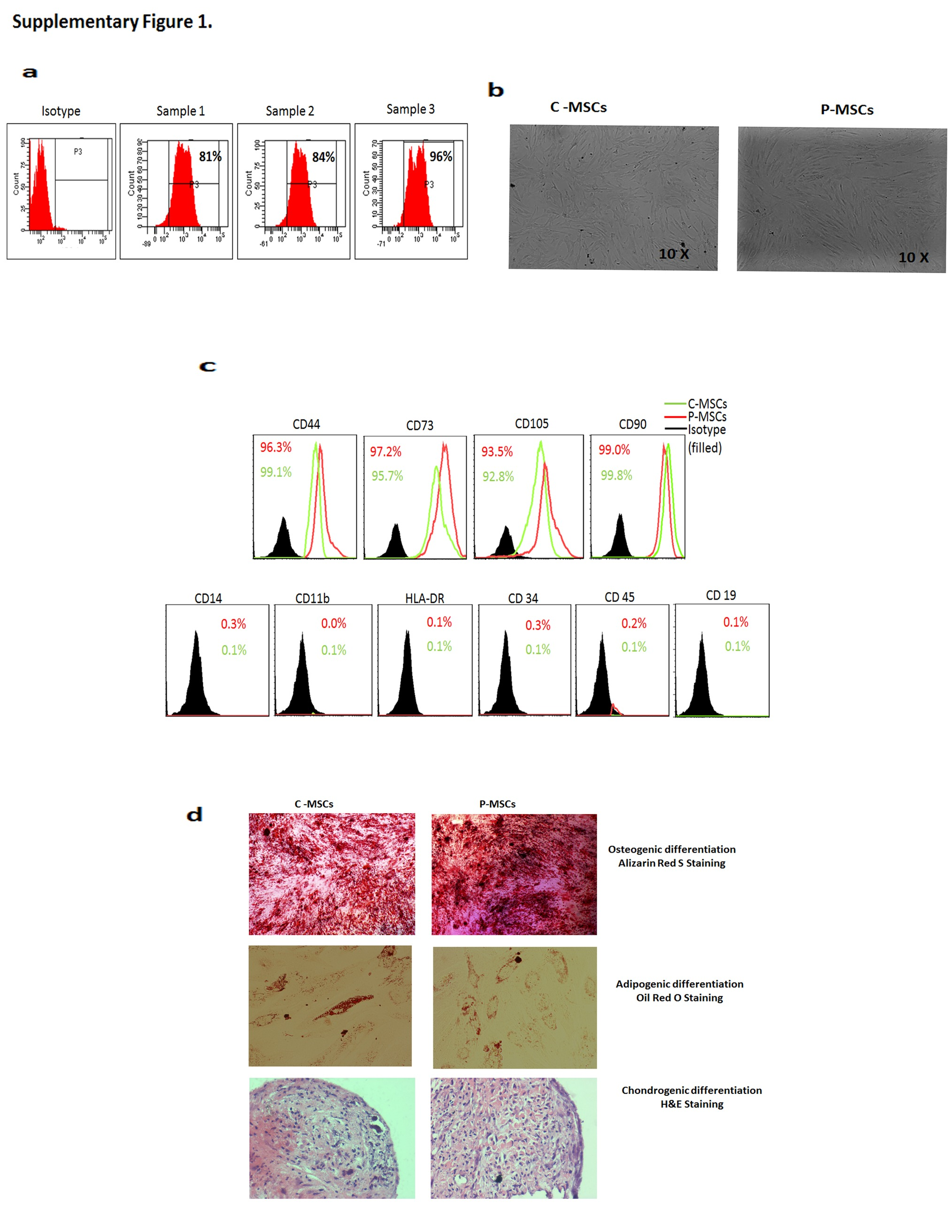

Supplement: S1 Fig — Isolated primary cells were subjected to phenotypic and functional characterization to check their purity prior to the experiment.(A)Representative histogram for 3 cord blood units to check their Purity after isolation from MNCs.(B) Fibroblastic morphology exhibited by C and P-MSCs as seen under phase contrast microscope(10X).(C) Overlays of histogram of representative samples of C and P-MSCs exhibiting expression of markers like CD44, CD90, CD73, CD105 and with no expression of CD45,CD34,CD14,CD19,CD11b and HLA-DR.(D) Upper panel represents osteogenic differentiation of MSCs by staining with alizarin red S. Middle panel is for adipogenic differentiation confirmed after lipid droplets stained by oil red o. Chondrogenic differentiation of C and P-MSCs as pellets confirmed with H&E staining. (TIF) [file pone.0165466.s001.tif]

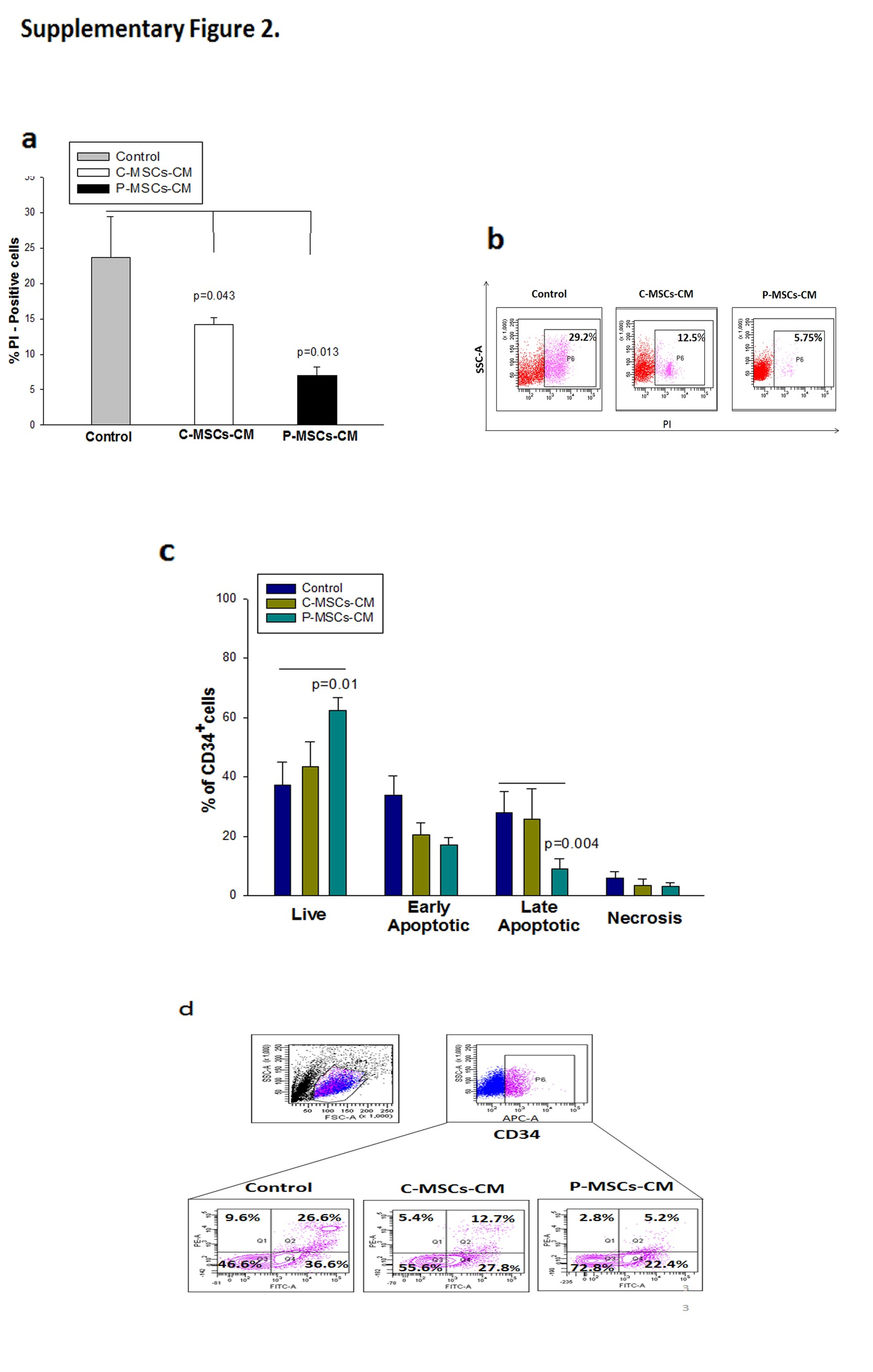

Supplement: S2 Fig — Expanded cells were subjected to Propidium iodide assay to check for the membrane damage and thus the viability.(A)The cells frozen with MSCs-CM had significantly lesser number of PI+ cells as compared to control.(B)The FACS profile of the representative samples depicting reduction in the percentage of PI+ cells in MSC-CM in comparison to control. (C) The level of apoptosis in the gated CD34+ cells was found to be significantly reduced in the P-MSCs-CM set. The % of viable cells was also higher p-MSCs-CM set. (D) FACS profile of representative sample depicting the distribution of revived CD34+cells at the various stages of apoptosis. Data is represented as Mean ± standard deviation from 3 different independent experimental sets. (TIF) [file pone.0165466.s002.tif]

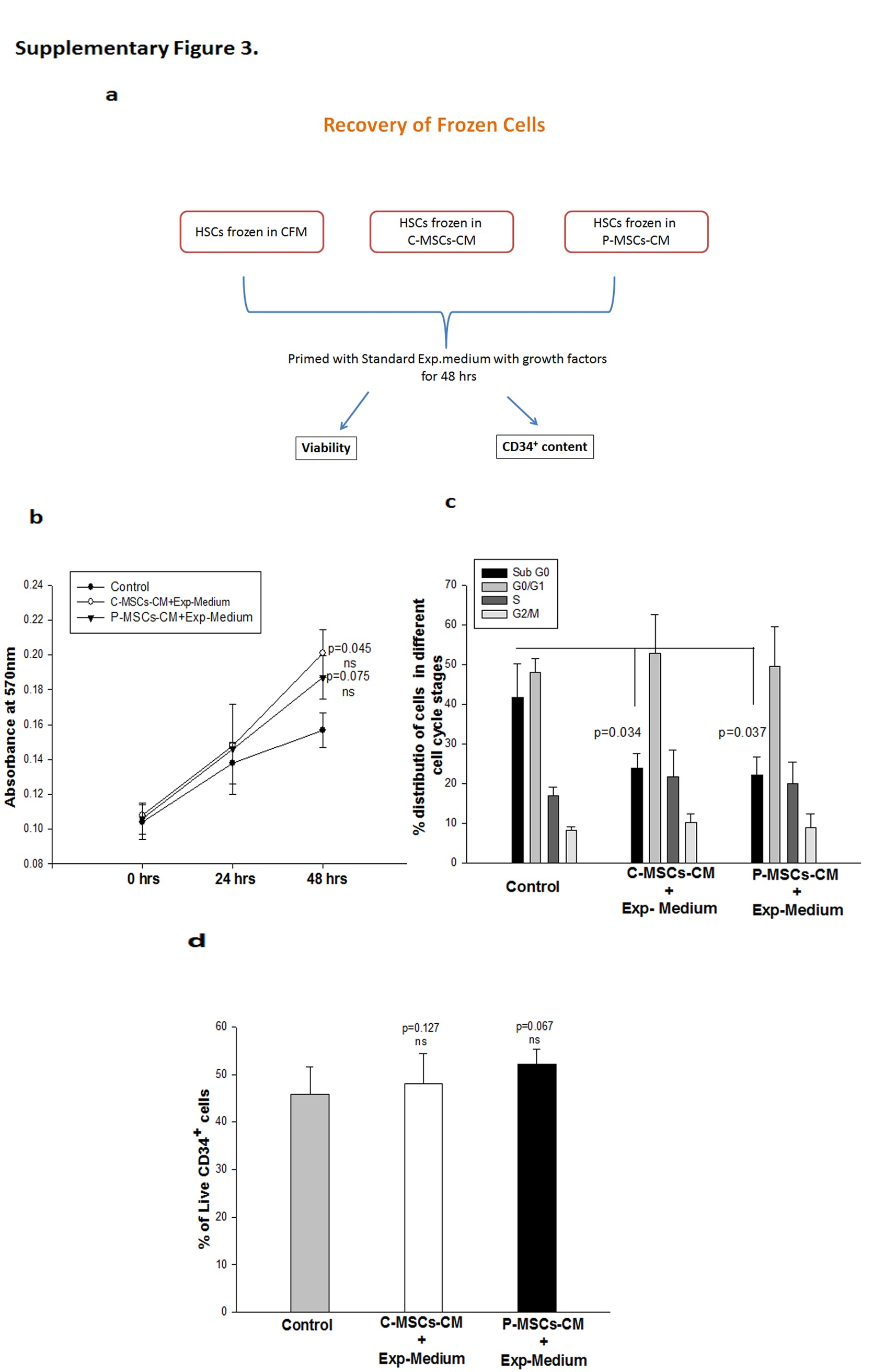

Supplement: S3 Fig — (A) Flow chart depicting the experimental design. (B)No significant difference in the proliferation was observed in the cells frozen in control or MSCs-CM and then re-cultured in Exp.medium. (C)The cell cycle analysis of these cells shows drastic reduction in the sub G0 phase with no change in the percentage of cells in the S and G2/M phase.(D)No difference was observed in the viability of CD34+ cells in all the three sets. Data is represented as Mean ± standard deviation from 3 different independent experimental sets. (TIF) [file pone.0165466.s003.tif]

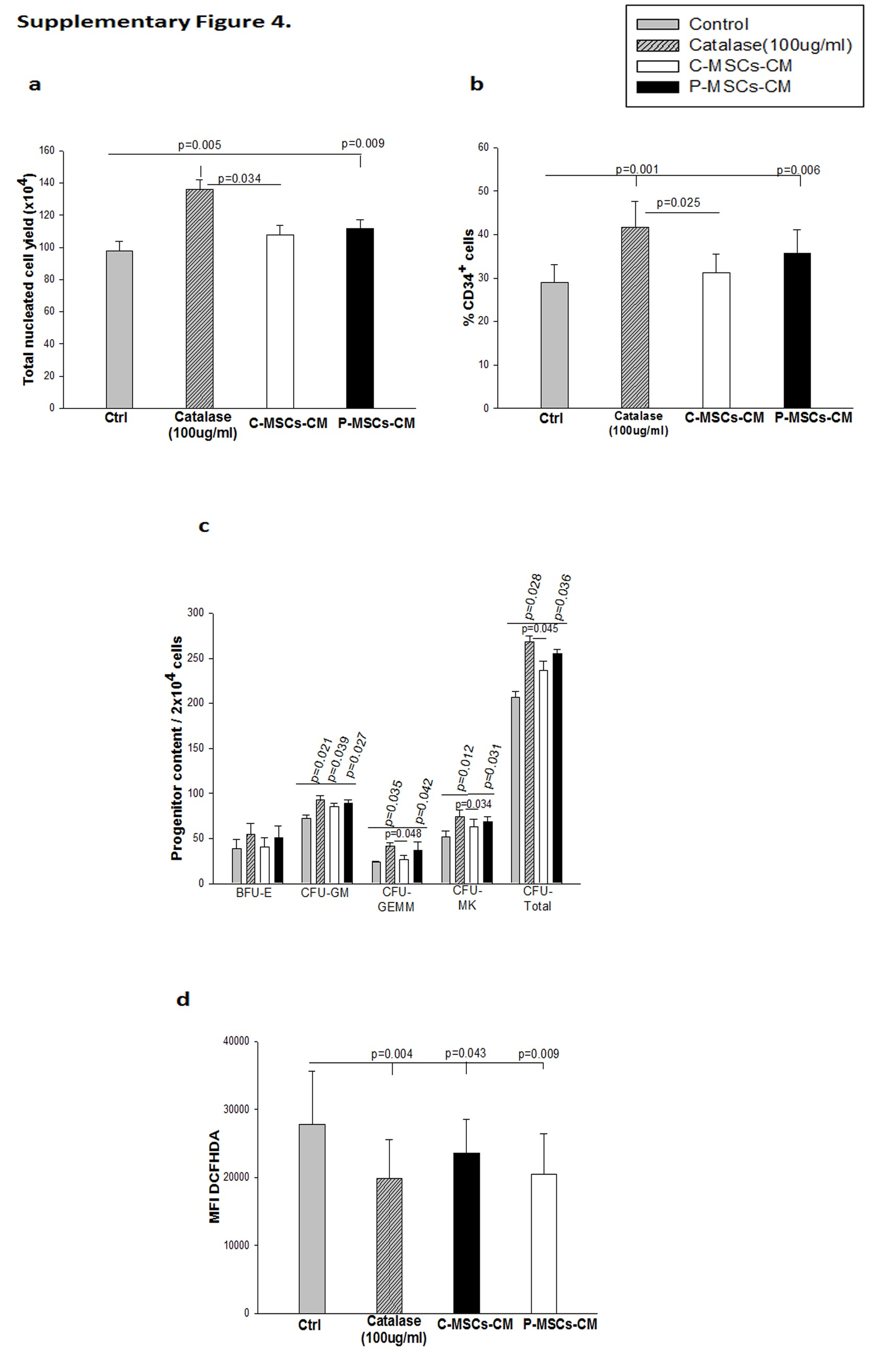

Supplement: S4 Fig — The expanded cells were frozen and primed with Control, Catalase (100μg/ml) as an additive in the CFM, C-MSCs-CM and P-MSCs-CM.(A) The cells frozen and re-cultured with catalase displayed maximum cell yield of total nucleated cells. The increase was also significant in the P-MSCs-CM set. (B)The CD34+ cells were also higher in catalase and P-MSCs-CM set.(C)Freezing and priming of expanded CD34+ cells with catalase resulted in to augmented clonogenecity of these cells. MSCs-CM set also displayed higher yield of blast-forming unit erythroid (BFU-E), granulocyte -monocyte(GM), granulocyte-erythroid-monocyte-megakaryocyte (GEMM)and Megakaryocytes (MK) colonies (d)Drastic reduction in total cellular ROS was seen in all the three sets as opposed to control set. Data is represented as Mean ± standard deviation from 3 different independent experimental sets. (TIF) [file pone.0165466.s004.tif]
